# Methanol poisoning and long term sequelae – a six years follow-up after a large methanol outbreak

**DOI:** 10.1186/1472-6904-9-5

**Published:** 2009-03-27

**Authors:** Raido Paasma, Knut Erik Hovda, Dag Jacobsen

**Affiliations:** 1 Department of Anesthesiology and ICU, Foundation Pärnu Hospital, Pärnu, Estonia; 2 Department of Acute Medicine, Ullevaal University Hospital, NO-0407, Oslo, Norway

## Abstract

**Background:**

Mass poisonings with methanol are rare but occur regularly both in developed and in developing countries. Data from the poisoning episodes are often published, but follow-up-data is scarce. We therefore conducted a six year follow-up study after the large methanol outbreak in Estonia in September 2001.

**Methods:**

Surviving victims from the outbreak were contacted and invited to an interview and a clinical evaluation by an ophthalmologist and a physician. The patients that failed to respond were searched for in the Estonian Register of Population and through their General Practitioner.

**Results:**

During the outbreak in 2001, 86/111 hospitalized patients survived: 66 without sequelae (Group I) and 20 with sequelae (Group II). Six years later, 26/86 were dead, 33/86 could not be tracked down, and so only 27/86 of these were followed up and examined: 22/66 of the patients in Group I, and 5/20 in Group II were found and examined. From Group I, 8/22 were identified with new neurological impairment and 8/22 with new visual disturbances after discharge. From Group II, visual disturbances (n = 4) and neurological impairment (n = 3) were still present in all patients. Among the 26 dead, 19 were from Group I, and seven were from Group II. Alcohol intoxication was the most frequent cause of death (7/26).

**Conclusion:**

All sequelae were still present six years after the initial poisoning suggesting that these were irreversible damages. On follow-up, apparently new neurological and visual complications were identified in 36% and 36%, respectively. 35% of the patients initially discharged with sequelae and 29% discharged without were dead six years later; 27% of them from alcohol intoxication.

## Background

Methanol is metabolized by the enzyme alcohol dehydrogenase (ADH) in the liver, via formaldehyde to formic acid, the latter being responsible for the adverse effects seen in methanol poisoning. The toxicity evolves from a combination of the metabolic acidosis (H^+ ^production) and an intrinsic toxicity from the formate anion itself [1,2]. Treatment consists of buffer such as sodium bicarbonate to correct metabolic acidosis and antidote to inhibit metabolism of methanol to its toxic metabolite, formic acid [3,4]. If necessary, haemodialysis is supplied to further correct the acidosis, and remove both methanol and formate [1,3,5-7]. In addition, folinic acid may enhance the endogenous metabolism of formate [2]. Methanol poisonings occur as isolated episodes caused by accidental or intentional ingestion, or epidemics. In the latter situation a large number of victims are often reported [8-10].

**Figure 1:**
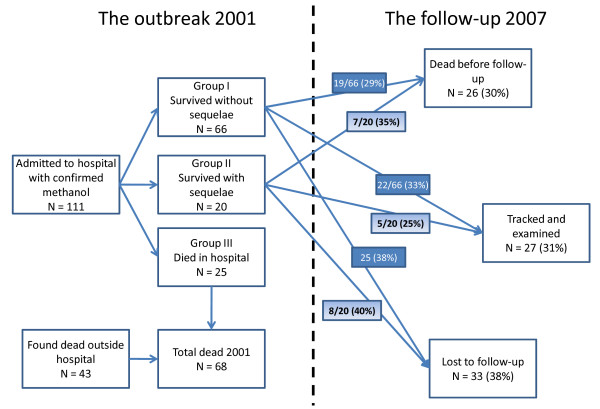
**Overview of the methanol outbreak in Estonia in 2001 and the follow-up study in 2007**.

In September 2001, illegal spirits containing 50–100% methanol were sold and consumed in the western part of Estonia in the Pärnu region. Out of a total of 147 patients admitted to hospital with suspicion of methanol poisoning, 36 did not have detectable serum methanol on admission, leaving a total of 111 hospitalized patients with verified methanol exposure. A total of 68 patients died: 25 in the hospital, whereas 43 were found dead from methanol poisoning outside the hospital. Thus, methanol poisoning was verified in a total of 154 patients (Figure 1). Patients were mainly treated with NaHCO_3 _infusion, ethanol infusion, dialysis and mechanical ventilation [9].

There is a lack of follow-up studies on methanol poisoned patients. The only literature found on the topic was the work done by Roe in 1943, where the visual disturbances in methanol poisoned patients were followed the weeks after discharge [11]. To evaluate the general outcome, the mortality, the visual disturbances and the neurological impairment after a methanol outbreak, we performed a follow-up study six years after the initial incident.

## Methods

Surviving victims from the methanol outbreak in 2001 in Pärnu, Estonia, were traced through hospital records from the former outbreak, and invited by letter and telephone to an interview and a clinical evaluation. The patients that failed to respond were searched for through the Estonian Register of Population and their general practitioners. The patients were after the 2001 outbreak classified into three groups: Those who survived without sequelae (Group I), those who survived with sequelae (Group II) and those who died (Group III). There were 66 patients in Group I, 20 patients in Group II, where visual disturbances and neurological impairment was the most common sequelae, and 25 patients in Group III [9].

The patients answered a questionnaire regarding their history of former diseases before and after the incident in 2001, as well as diseases at present (including diabetes). They were questioned about their drinking habits before and after the initial outbreak in 2001. Further, all prescriptional medications used in 2001 and at present were registered.

The medical and the neurological examination were performed by a physician, whereas the ophthalmological examination was performed by an ophthalmologist: All findings were compared to the clinical status at discharge six years earlier. The data were categorized in a descriptional manner, hence no further statistical analyzes were performed.

The study was approved by Tallinn Medical Research Ethics Committee, following an individual agreement from all participating patients.

## Results

After the outbreak in 2001, there were 86 survivors (66 without sequelae – Group I, and 20 with sequelae – Group II). Six years later, 26 (30%) were dead (5 females and 21 males), 33 (38%) were lost to follow-up, and so only 27 (31%) were tracked and examined (all of the latter agreed to participate in the study): 22/66 (33%) of the patients in Group I (6 females and 16 males), and 5/20 (25%) in Group II (1 female and 4 males) were among these (Figure 1).

Since 2001, 8/22 in Group I – the survivors without sequelae – were identified with neurological impairment and 8/22 with visual disturbances (Table 1 [see Additional file 1]). The latter complications were all revealed by an ophthalmologist. None of the patients had diabetes. In Group II – the survivors with sequelae – 18/20 had visual disturbances at discharge in 2001. Six years later 4/18 were tracked and examined, visual disturbances were still present in all of them.

The most common visual disturbances present at the time of follow-up (both groups-present at discharge and apparently acquired after discharge) were: Optical nerve atrophy, temporal pallor of the optic nerve head, visual field defects, and loss of visual acuity (severe to deep blindness). Further, 3/20 in Group II had neurological impairment on discharge from hospital six years earlier and this was still present, whereas one additional patient had developed neurological impairment six years after the initial discharge (Table 1 [see Additional file 1]).

The clinical symptoms among the patients with neurological impairment were the same in both groups (present at discharge and acquired after discharge): Polyneuropathy and encephalopathy (from light to severe), ataxic gait (unstable walking), Romberg test positive (meaning that they cannot stay upright with their eyes closed), or sensory loss on the distal part of the legs (Table 1 [see Additional file 1]).

Among the 26 who died after discharge from the poisoning episode in 2001, 19 were from Group I (survivors without sequelae) and 7 were from Group II (survivors with sequelae) (Figure 1). Alcohol intoxication (unknown kind of alcohol and origin) was the most frequent cause of death (7/26), whereas other causes of death were cardiac reasons (6/26; including cardiomyopathy (n = 2) and myocardial infarction (n = 2)), trauma (3/26), carbon monoxide-poisoning (3/26) or pneumonia (2/26).

**Figure 2:**
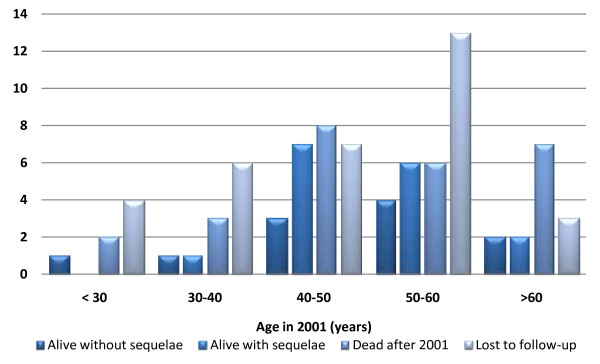
**Status on follow-up after six years (patients grouped according to their age in 2001)**.

The status on follow-up after six years among the different age-groups is shown in Figure 2: Only 11 patients were tracked and found alive without sequelae. The mortality was relatively evenly distributed in the different age groups (approximately 30%), except from the age group >60 years, where 50% were dead after six years. There were relatively more patients lost to follow-up in the two youngest age-groups (approximately 55%), vs. 45% among the 50–60 year olds, and 21–28% among the rest.

Only a few of the patients who drank alcohol on a regular basis before the methanol outbreak reduced their drinking habits after the incidence: Among the patients from Group II, 1/5 was still drinking alcohol in the same amount and frequency as he did before the initial outbreak in 2001, whereas 2/5 had reduced their alcohol consumption and 2/5 had stopped using alcohol completely.

Among the patients from Group I, 10/22 developed sequelae *after *discharge (6 both VD and neurological impairment (NI), 2 VD and 2 NI): There were fewer patients who stopped drinking in this group (1/10) vs. the ones who did not develop sequelae (5/12). 3 patients were still drinking alcohol in the same amount and frequency as before the methanol poisoning among both the ones who acquired sequelae after discharge, and those who did not. Regarding their "quality of life" and disability, we found that 6/27 of those tracked six years later now needed help in their activities of daily living.

## Discussion

Despite efficient treatment [3], methanol poisoning has a high morbidity and mortality [2,10,12,13]. Some patients with methanol poisoning will probably remain unknown in a large outbreak, either having lesser symptoms and never being examined, or dying without the diagnosis of methanol poisoning being suspected [10]. The prognosis depends on the degree of metabolic acidosis [2,10,14]. Different findings on admission has been suggested as poor prognostic factors, e.g. high S-methanol concentration [8,13], although others do not find this [10,12,15]. Coma on admission is generally considered a poor prognostic sign [8,10,13], and recently, increased pCO_2 _in the severely acidotic patient (lack of compensatory hyperventilation) is suggested as a new marker [9,10]. The most frequent sequelae found at discharge are visual disturbances and neurological impairment of some kind.

This follow-up study was performed to evaluate the course of the patients in the aftermath of a methanol poisoning outbreak. We found that visual disturbances present at discharge was still present six years later and were of the same magnitude, supporting the findings from 1943 by Roe [11]. In 8/22 patients discharged without visual disturbances in 2001, these complications were found six years later. Patients were all evaluated by an ophtalmologist, who considered the methanol poisoning to be the likely cause: The main findings included optical nerve atrophy, temporal pallor of the optic nerve head, concentrical reduction of the visual field, and loss of visual acuity (Table 1 [see Additional file 1]). One likely explanation is that many of those patients were discharged without a clinical evaluation by an ophthalmologist. It may also partly be explained by a gradual development of symptoms over time, making them symptomatic and possible to measure, rather than a pure late onset complication. Regardless, this raises the question whether this phenomenon is an under-reported feature also in other outbreaks.

Neurological impairment was generally increased during these six years: As many as 8/22 patients discharged without neurological impairment developed neurological complications: The most likely reason for that is the continuous use of excess alcohol: There were fewer patients who stopped drinking among the patients who developed the neurological impairment.

There was a high death toll (30%), also among the patients discharged alive from the outbreak in 2001. The mortality was slightly higher among the ones in Group II (discharged with sequelae) (35%), compared to the ones in Group I (29%). The causes of death was dominated by alcohol intoxication (27%), cardiac diseases (23%), and traumas (19%), indicating that the this is an exposed and vulnerable group: In 2001, all age groups were represented from below 30 to above 60 years old, but there was an overrepresentation of frequent drinking, middle-aged males [9]. The fact that only 12 patients was tracked and found alive without sequelae six years later further supports this argument.

Generally, there was a high mortality in all age groups (approximately 30%), with a higher mortality (50%) among the oldest ones (>60 years) as expected. More patients were lost to follow-up among the youngest patients, but no obvious reasons for this were found. Unfortunately, the drinking habits of many of the patients did not improve: A lot of them were still using excessive amounts of alcohol despite the serious methanol poisoning incident. This indicates that individuals affected by a methanol outbreak may benefit from counselling and other efforts aimed at reducing their alcohol abuse.

## Conclusion

The morbidity and mortality of methanol poisoning remains high, even after discharge from hospital. Likely reasons for this are the patients themselves often being a high-risk group with a high alcohol-attributable burden. In this population, the death toll among the survivors from the outbreak in 2001 was high even within the youngest age-groups. Sequelae from methanol poisoning are likely to persist or even progress (both visual disturbances and neurological impairment): Visual disturbances may be a feature developing over time, hence be an under-estimated feature in methanol poisoned patients in general. The risk of developing neurological impairment seems to be higher if drinking habits are not changed.

## Competing interests

The authors declare that they have no competing interests.

## Authors' contributions

RP participated in the planning of the study, drafted the manuscript, examined the patients, and coordinated the collection of the data. KEH participated in the planning of the study, co-wrote the manuscript and supervised the work. DJ participated in the planning of the study, co-wrote the manuscript and supervised the work. All authors participated in revising the manuscript, and have read and approved the final version.

## Pre-publication history

The pre-publication history for this paper can be accessed here:



## Supplementary Material

Additional file 1 The patients with visual disturbances (VD) or neurological impairment (NI) in 2007. Click here for file
