# Supplementary material for: Methanol poisoning and long term sequelae – a six years follow-up after a large methanol outbreak
Source: BMC Clin Pharmacol. 2009 Mar 27;9:5. doi: 10.1186/1472-6904-9-5 (PMC2667428; doi:10.1186/1472-6904-9-5)
Supplement: Additional file 1 — The patients with visual disturbances (VD) or neurological impairment (NI) in 2007. [file 1472-6904-9-5-S1.doc]

|  | | | | | Visual disturbances (VD) in 2007 | | | | Neurological impairment (NI) in 2007 | | | | |
| --- | --- | --- | --- | --- | --- | --- | --- | --- | --- | --- | --- | --- | --- |
| Patient | Gender & age (2007) | Group in 2001 | VD in 2001? | NI in 2001? | Optical nerve atrophy | Temporal pallor of the optic nerve head | Visual fields defects | Loss of visual acquity | Poly-neuropathy | Encephalopathy | Ataxic gait (Unstable walk) | Romberg positive | Sensory loss  of distal parts of the legs |
| 1 | F68 | I |  |  |  |  | + |  |  |  |  |  |  |
| 2 | F62 | I |  |  |  |  |  |  |  | + |  |  |  |
| 3 | M41 | I |  |  |  | + | + |  |  | + | + | + | + |
| 4 | M47 | I |  |  |  |  | + |  |  | + | + |  |  |
| 5 | M51 | I |  |  |  | + |  |  |  |  |  |  |  |
| 6 | M65 | I |  |  | + | + | + | + | + | + |  | + |  |
| 7 | M68 | I |  |  |  |  |  |  | + | + | + |  | + |
| 8 | M53 | I |  |  |  |  | + |  | + | + | + | + |  |
| 9 | M52 | I |  |  | + |  |  |  | + |  | + | + | + |
| 10 | M48 | I |  |  |  | + | + |  |  | + | + |  |  |
| 11 | M65 | II | + | + |  | + | + | + | + | + | + |  | + |
| 12 | F55 | II | + | + | + |  |  | + | + | + | + | + | + |
| 13 | M55 | II | + |  | + |  |  | + |  |  |  |  |  |
| 14 | M47 | II |  | + |  |  |  |  | + | + | + |  | + |
| 15 | M59 | II | + |  | + |  | + | + | + | + | + |  | + |

**Table 1** The patients with visual disturbances (VD) or neurological impairment (NI) in 2007
